# Using Cochlear Microphonic Potentials to Localize Peripheral Hearing Loss

**DOI:** 10.3389/fnins.2017.00169

**Published:** 2017-04-04

**Authors:** Karolina K. Charaziak, Christopher A. Shera, Jonathan H. Siegel

**Affiliations:** ^1^Caruso Department of Otolaryngology, Keck School of Medicine, University of Southern CaliforniaLos Angeles, CA, USA; ^2^Roxelyn and Richard Pepper Department of Communication Sciences and Disorders, Hugh Knowles Center, Northwestern UniversityEvanston, IL, USA

**Keywords:** cochlear microphonic, electrophysiology, cochlea, acoustic trauma, hearing loss

## Abstract

The cochlear microphonic (CM) is created primarily by the receptor currents of outer hair cells (OHCs) and may therefore be useful for identifying cochlear regions with impaired OHCs. However, the CM measured across the frequency range with round-window or ear-canal electrodes lacks place-specificity as it is dominated by cellular sources located most proximal to the recording site (e.g., at the cochlear base). To overcome this limitation, we extract the “residual” CM (rCM), defined as the complex difference between the CM measured with and without an additional tone (saturating tone, ST). If the ST saturates receptor currents near the peak of its excitation pattern, then the rCM should reflect the activity of OHCs in that region. To test this idea, we measured round-window CMs in chinchillas in response to low-level probe tones presented alone or with an ST ranging from 1 to 2.6 times the probe frequency. CMs were measured both before and after inducing a local impairment in cochlear function (a 4-kHz notch-type acoustic trauma). Following the acoustic trauma, little change was observed in the probe-alone CM. In contrast, rCMs were reduced in a frequency-specific manner. When shifts in rCM levels were plotted vs. the ST frequency, they matched well the frequency range of shifts in neural thresholds. These results suggest that rCMs originate near the cochlear place tuned to the ST frequency and thus can be used to assess OHC function in that region. Our interpretation of the data is supported by predictions of a simple phenomenological model of CM generation and two-tone interactions. The model indicates that the sensitivity of rCM to acoustic trauma is governed by changes in cochlear response at the ST tonotopic place rather than at the probe place. The model also suggests that a combination of CM and rCM measurements could be used to assess both the site and etiology of sensory hearing loss in clinical applications.

## Introduction

The practical application of anticipated pharmacological and genetic treatments for hearing loss will require diagnostic tests that can differentiate between sites and etiologies of the damage. Cochlear microphonic (CM) potentials could aid the diagnosis of sensory hearing loss by revealing cochlear regions with impaired outer hair cells (OHCs). Here, we use an animal model to test whether a new approach to CM measurements allows for detection of a notch-type sensitivity loss resulting from the disruption of OHC function (i.e., moderate acoustic trauma).

The CM is an alternating-current (AC) potential created primarily by the mass receptor currents of OHCs following basilar-membrane (BM) movement (e.g., Dallos and Cheatham, 1976). Conventionally, CM is measured in the steady state as a response to pure-tone stimulation. Despite the use of a tonal stimulus that reaches peak excitation at a specific cochlear location, the CM has poor spatial resolution, as it constitutes a complex sum of potentials produced by all the cells excited by the BM traveling wave. Due to the rapid phase variation of the BM displacement near the characteristic-frequency (CF) place of the tonal stimulus, the currents from OHCs located in that active region tend to cancel and contribute little to the measured CM. As a result, the CM is dominated by contributions from OHCs located in the passive tail region of the BM excitation, where the phase varies little with location and currents sum constructively (Dallos, 1973). Furthermore, the CM depends on the position of the recording electrode relative to the CM sources: both electrical attenuation of the cochlear potentials with distance from the source as well as the spiral shape and complex electroanatomy of the cochlea can affect the measured response (e.g., von Békésy, 1951; Chertoff et al., 2012). Together, these factors limit the CM's place-specificity (i.e., the ability to assess the function of OHCs located near the CF place of the stimulus). A dramatic demonstration of this limitation comes from a classic study by Patuzzi et al. (1989b) in guinea pig. In the study, the ablation of the apical turn of the cochlea had little effect on the CM measured at the round window (RW) in response to a low-frequency tone that would normally have peaked near the apical end. These limitations have hindered the clinical application of the CM, which now serves primarily as a gross indicator of OHC function across the cochlea (e.g., Gibson and Sanli, 2007; Radeloff et al., 2012).

We suggest that the poor sensitivity of the CM to local changes in OHC activity might be overcome by exploiting the properties of cochlear two-tone suppression. Two-tone suppression is observed in the BM responses of a healthy cochlea when the response to one tone (probe) is reduced by the presence of another (suppressor) tone (e.g., Ruggero et al., 1992). The suppressor is believed to act locally, near its own CF place, by saturating the receptor currents of nearby OHCs (Geisler et al., 1990). Two-tone interactions can be also detected in the CM, although, unlike for the single-location BM responses, the secondary tone can result in both reduction as well as enhancement of the probe-tone CM (Legouix et al., 1973; Cheatham and Dallos, 1982; Nuttall and Dolan, 1991; He et al., 2012). Thus, in the context of CM measurements, we refer to this secondary tone as a “saturating tone” (ST) to avoid the implicit assumption that, as in classic BM measurements, a secondary tone leads exclusively to a “suppressed” probe-tone response. The complex behavior of CM two-tone interactions has been explained as the result of changes in the spatial summation pattern of the voltage sources along the BM, which can produce CM enhancement (Nuttall and Dolan, 1991). However, near its own CF place, the ST presumably acts primarily as a “suppressor” of local CM sources (i.e., it saturates the transducer currents of nearby OHCs), as suggested by CM measurements from within the organ of Corti (Nuttall and Dolan, 1991). Thus, it may be possible to extract information about local OHC health by evaluating only the CM component(s) affected by the ST. In theory, this can be accomplished by deriving the complex difference between the probe-tone (PT) CMs obtained both with and without the ST; that is, by measuring the “residual CM” (rCM; Siegel, 2006). Ideally, the rCM represents contributions from the subpopulation of CM sources excited by the probe and suppressed by the ST near its CF place in the cochlea. It may therefore be possible to localize regions with malfunctioning hair cells by varying the probe and the ST frequencies together across the hearing range (e.g., at a constant ratio). In such a case, we expect the rCM to decrease in magnitude when the excitation pattern of the ST reaches the damaged region. A similar method has been successfully employed in otoacoustic emission (OAE) measurements for detecting local changes in cochlear sensitivity (e.g., Martin et al., 2010).

Here, we assess the ability of the rCM measured at the round window to detect a notch-type moderate loss of sensitivity in chinchillas. We induce the change in sensitivity via short exposure to an intense tone, as such trauma has been shown primarily to affect OHC function, resulting in diminished BM nonlinearity (e.g., Pickles et al., 1987; Puel et al., 1988; Davis et al., 1989; Ruggero et al., 1996; Nordmann et al., 2000; Chertoff et al., 2014). We test the hypothesis that rCM represents a response from sources located near the CF place of the ST in the cochlea by obtaining measurements at varying *f*_ST_/*f*_PT_ ratios (ranging from ~1 to 2.6) both before and after inducing the acoustic trauma. If rCM indeed represents responses from CM sources located near the ST place, rCM will drop in level when the ST frequency—but not necessarily the probe-tone frequency—matches the frequency of the sensitivity loss.

Lastly, to test the above prediction and to improve the interpretation of the data, we present a simple phenomenological model of CM generation and two-tone interactions based on published BM data from chinchillas. With this study, we aim to demonstrate that a new approach to CM measurements makes it possible to extract place-specific information about OHC function, thereby enhancing the diagnostic utility of electrocochleography.

## Methods

### Animal preparation

Most of our methods have been described previously (Charaziak and Siegel, 2014, 2015). Adult chinchillas were anesthetized with ketamine hydrochloride (20 mg/kg, injected subcutaneously), followed by Dial (diallylbarbituric acid) in urethane (initial doses 50 and 200 mg/kg, respectively) with additional doses (20% of the initial one) given as necessary. The animals were trachetomized, but forced ventilation was not used. The pinna and the lateral portion of the external auditory meatus were removed. The tip of the microphone probe system was placed near the tympanic membrane (~2 mm) and the probe was sealed with impression material. The bulla was opened, the tensor tympani was sectioned, and a silver-ball electrode was placed on the round window. The reference electrode was inserted in the skin of the contralateral ear, and the ground electrode was attached to the head holder. The rectal temperature was kept at ~37°C. The preparation was monitored via repeated recordings of distortion-product OAEs (not reported), CAP thresholds, and CMs throughout the duration of data collection (~9 h). The data collection involved experiments that were a part of another study (Charaziak and Siegel, 2015; Siegel and Charaziak, 2015). Experimental protocols were approved by the Animal Care and Use Committee of Northwestern University.

### Instrumentation

All measurements were carried out in an electrically shielded sound-attenuating booth. Stimulus waveforms were generated and responses acquired and averaged digitally using 24-bit sound card (Card Deluxe-Digital Audio Labs; sampling rate 44.1 kHz) controlled with EMAV software ver. 3.24 (Neely and Liu, 2015). The round-window (RW) electrode signal was differentially amplified (40 dB), band-pass filtered (0.1–30 kHz), and corrected for the acoustic delay of the sound-delivery system, as well as for the delay of the preamplifier filter. The output of the probe microphone (Etymōtic ER-10A) was amplified (20 dB), high-pass filtered (0.15 kHz), and corrected for acoustic delays and mic sensitivity (Siegel, 2007). The stimuli were presented either via two modified Radio Shack RS-1377 Super Tweeters (for CAP/OAE/CM measurements) or via Fostex FT17H Horn Super Tweeter (for tonal overexposures) coupled via plastic tubing to the probe-microphone system. The speakers were grounded and shielded with heavy gauge steel boxes to minimize electrical and magnetic radiation. Potential contamination of the CM signals from the speakers was below the system's noise floor for all stimulus conditions. The stimulus levels were calibrated *in situ* to maintain a constant pressure level at the inlet of the probe microphone near the eardrum.

### Measurements and analyses

The RW signal was measured in response to stimulation with pure tone(s) (~1.57 s duration, including 10-ms onset/offset ramps). The stimuli were presented in recording blocks, each consisting of four conditions: probe tone (PT) alone, PT + near-probe-frequency saturating tone (ST), PT + high-frequency ST, PT + both STs (not reported). The four conditions were presented in sequence (with ~200 ms gaps in between conditions), and the ST and PT were always delivered via separate sound sources. Each condition was immediately repeated and the responses were stored in separate buffers (A and B). The two response buffers were averaged ($\frac{\text{A}+\text{B}}{2}$) and subtracted ($\frac{\text{A}-\text{B}}{2}$) from each other to obtain estimates of either the CM or the noise amplitude at the frequency of the probe (via Fast Fourier transform), respectively. In both cases, the first and the last 46.4 ms of the response buffer, were skipped to prevent contamination from responses to onset and offset transients (e.g., CAP). The probe tone (30 dB SPL, *f*_PT_: 0.33–10 kHz in steps of 86 Hz), and near-probe ST (55 dB SPL, *f*_PT_–43 Hz, *f*_ST_/*f*_PT_ ≈ 1) conditions were fixed, while a different, higher frequency ST (55 dB SPL, *f*_ST_/*f*_PT_ = 1.2, 1.4, 2.1, or 2.6) was used for each recording block (four in total). For the *f*_ST_/*f*_PT_ = 2.6 condition, the value of *f*_PT_ was limited to 8 kHz to keep *f*_ST_ below the Nyquist frequency. For convenience, we abbreviate the various *f*_ST_/*f*_PT_ ratio conditions as ST1, ST1.2, and so on, where the number gives the value of *f*_ST_/*f*_PT_. The rCMs were calculated as vector differences between RW responses to the PT alone and PT + ST presentations for any given PT (e.g., rCM_ST1_ = CM_PT_–CM_PT+ST1_). For comparison, the response to the PT alone (i.e., the “conventional” CM) was also evaluated. The same set of measurements was obtained before and after inducing the acoustic trauma. The PT alone and ST1 conditions were retested together with each higher-ST condition and were thus used to evaluate the stability of the preparation (in terms of CM and rCM_ST1_). Unless stated otherwise, the probe-alone and ST1 data reported here were collected in the block of stimuli used to measure the ST2.1 condition.

Although CM measured at the RW may contain contributions from sources other than OHC receptor currents (see Discussion), we adhere to the terminology used previously in the literature and refer to the RW cochlear potential synchronized with the stimulus collectively as CM.

### Tonal overexposure

The acoustic trauma was induced by exposure to an intense 3-kHz tone (100–106 dB SPL) presented in 4-min time blocks until at least 30-dB sensitivity loss was achieved at and/or above 4 kHz as monitored with CAP thresholds (criterion response: 10 μV, see Charaziak and Siegel, 2015 for measurement details). Reaching the target CAP threshold elevation required total exposure durations ranging from 4 to 16 min across the animals (*n* = 4). When possible, CAP thresholds were re-measured at the termination of the experiment. Because the tone-pip-evoked CAP represents responses from auditory-nerve fibers innervating a region around the CF place of the stimulus (Teas et al., 1962; Özdamar and Dallos, 1978), changes in CAP thresholds faithfully reflect changes in local BM sensitivity following acoustic trauma (Ruggero et al., 1996). Thus, for the purposes of this study we equate the frequency-specific shifts in CAP thresholds with place-specific decreases in OHC-dependent gain.

## Results

In the following sections, we present data obtained in four chinchillas. In these four animals, the repeated measures of CM and rCM_ST1_ usually varied by <5 dB within pre- or post-exposure measurement blocks, except for run ST2.6 for animal E23 (last run in the post-exposure block; changes > 20 dB). Thus, the ST2.6 data for E23 were excluded from the analysis. Two out of four animals had initial notch-like elevations in their CAP thresholds that were either preexisting or induced by the surgery (~30 dB near 5.6 kHz for G03, and ~25 dB near 10 kHz for E23). The pre-existing threshold shift abolished neither the distortion-product OAEs evoked with low or moderate level tones nor the CM and rCM, suggesting that functioning OHCs were still present in the affected regions. Because we were interested in detecting changes in CM and rCM due to experimentally induced CAP threshold shifts, these animals were not excluded from the analysis.

### Effect of the acoustic trauma on CM and rCM

**Figure 1:**
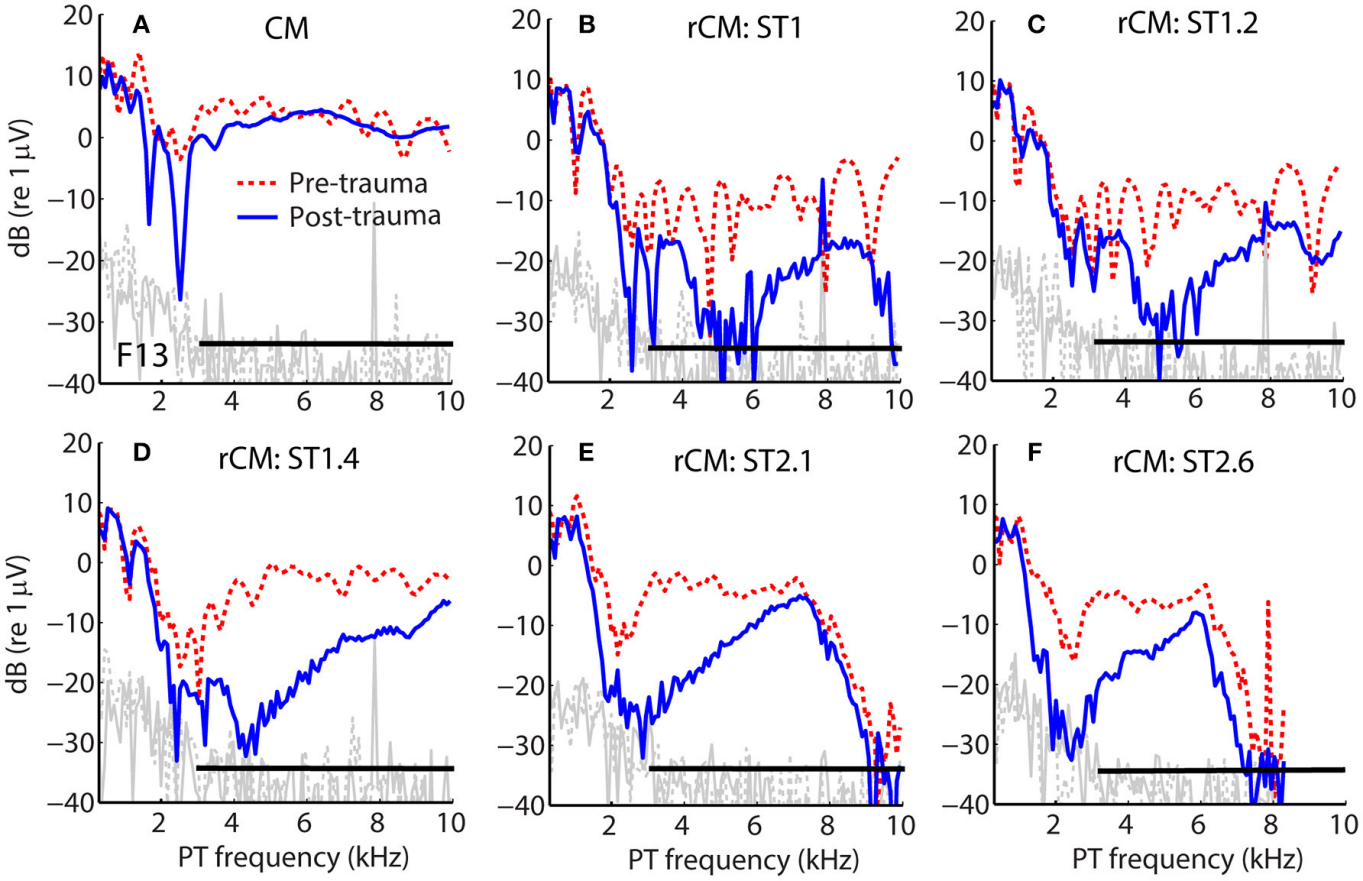
**Example of CM (A)** and rCM **(B–F)** levels measured in a chinchilla before (dashed red) and after (solid blue) inducing an acoustic trauma. The black horizontal bar marks the frequency range with CAP sensitivity loss > 20 dB (3–11 kHz, with maximal shift at 6.3 kHz of 32 dB; also see Figure 2B). Noise floors are shown in gray.

Figure 1 shows examples of CM responses collected before and after the acoustic trauma for a representative animal (F13). Although the acoustic trauma had relatively little effect on CM levels (Figure 1A, dotted red vs. solid blue), rCM levels decreased by up to ~20 dB (B–F). The frequency range of the largest decreases in rCM level varied across the ST conditions, shifting toward lower probe frequencies at higher *f*_ST_/*f*_PT_ ratios.

**Figure 2:**
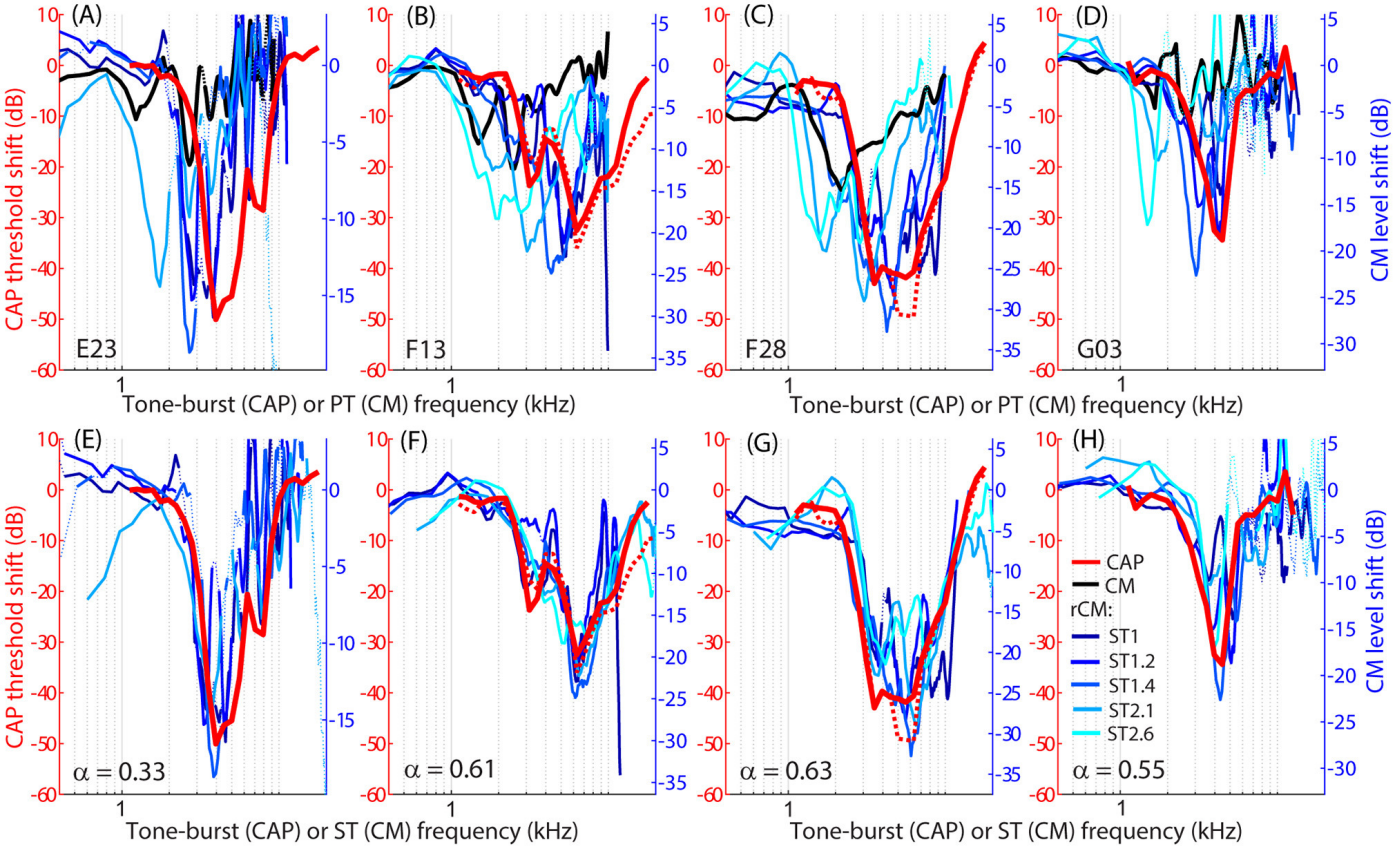
**Shifts in CM (black) and rCM levels (shades of blue, see legend in H) compared to shifts in CAP thresholds (red) resulting from an acoustic trauma in four chinchillas (columns)**. Note that CAP threshold shifts are plotted on left *y*-axes while CM and rCM changes are plotted on right *y*-axes. The CM *y*-axis was scaled for each animal in an iterative process until the root-mean-square error between rCM level shifts (plotted against ST frequency) and CAP shift was minimized. The scaling factors α are listed on the bottom panels (i.e., rCM or CM level shift equals to α x CAP shift in dB). Data with pre-exposure SNR <6 dB are shown with dotted lines. The CM and rCM shifts were gently smoothed (moving average). In panels **(A–D)**, the rCM changes are plotted against the probe tone (PT) frequency and in **(E–H)** against the saturating tone (ST) frequency. For animals F13 and F28, CAP thresholds were re-measured at the end of the experiment to confirm the stability of the threshold shift (red dotted).

**Figure 3:**
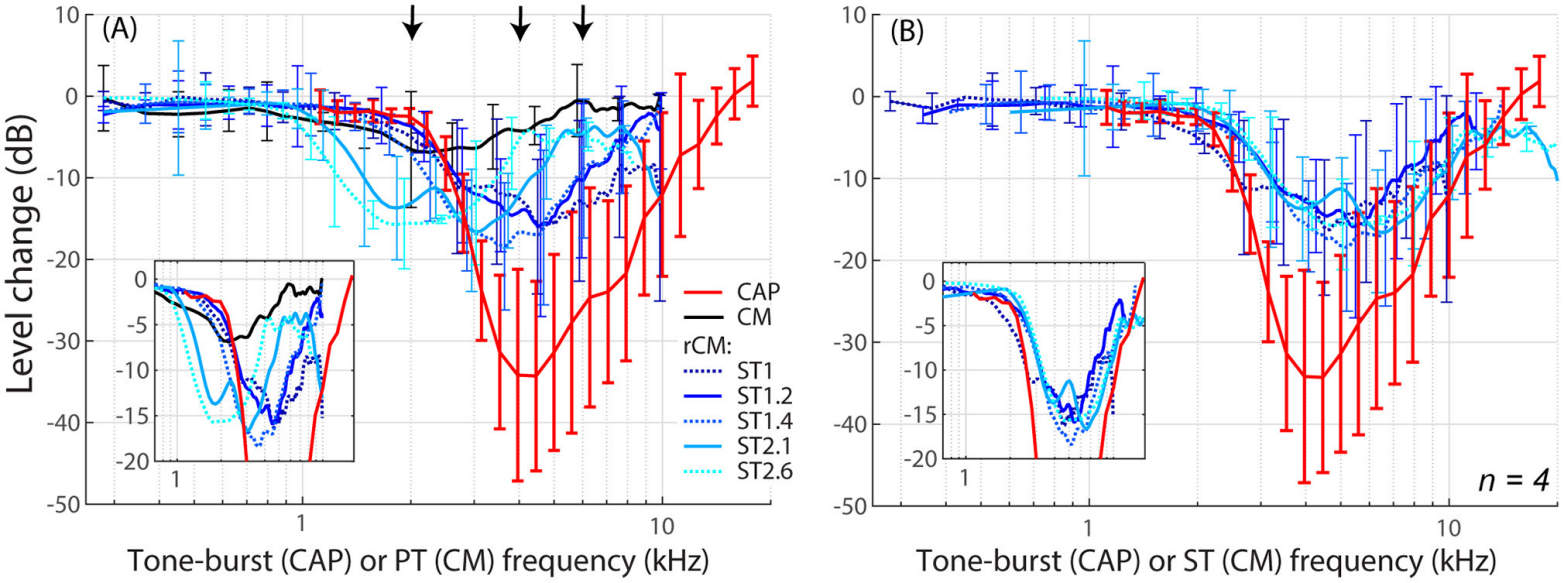
**Mean shifts in CM and rCM levels (shades of blue, see legend in A) compared to mean shifts in CAP thresholds (red) resulting from an acoustic trauma in four chinchillas**. In panel **(A)** the rCM changes are plotted against the probe tone (PT) frequency and in **(B)** they are plotted against the saturating tone (ST) frequency. The error bars represent standard deviation of a mean (for the CM data error bars are shown every ~0.4 octave). Only data with pre-exposure SNR > 6 dB were included in the average (see solid lines in Figure 2), and the grand average was gently smoothed (moving average). The black arrows in A indicate frequencies at which data were compared to the model (Figures 5, 6). The insets in each panel show the same data plotted with the error bars omitted to emphasize the alignment with the CAP data.

The group data are shown in Figures 2A–D, where trauma-induced changes in the CAP thresholds, and CM and rCM levels are plotted against the probe frequency for each animal. The corresponding average data are shown in Figure 3A. The exposure to an intense 3-kHz tone created a ~35 dB (32–50 dB range) notch-type sensitivity loss centered at 4 kHz (red) that could be attributed to malfunctioning OHCs (e.g., Saunders et al., 1991; Ruggero et al., 1996). Despite substantial loss of sensitivity, CM levels decreased on average by no more than ~7 dB (Figure 3A, black; 7–14 dB range, Figures 2A–D), with the largest change occurring at frequencies 0.6–0.7 octaves lower than the frequency of maximal shift in CAP thresholds. If the CM is dominated by potentials from OHCs located in the passive tail region of the BM excitation, the observed drop in CM level is consistent with decreased OHC transduction currents in the traumatized region (Patuzzi et al., 1989a; Nakajima et al., 2000). In contrast, for any ST condition tested, rCM level decreased on average by ~15 dB following the trauma. The range of affected probe frequencies varied systematically with the ratio *f*_ST_/*f*_PT_: The higher the ratio, the lower the frequency of the maximal shift (Figures 2A–D, 3A also see inset). Typically, a 1 dB of CAP threshold shift resulted in ~0.6 dB of rCM level shift (see values of the scaling factor α in Figures 2E–H; see caption for details).

When the changes in rCM levels are plotted against the ST frequency (Figures 2E–H, 3B also see inset), the range of affected frequencies coincides well with the range over which loss of sensitivity was observed (blue lines vs. red). This result supports our hypothesis that rCM originates predominately near the CF place of the ST, rather than the PT. Also note that if the rCM measures changes in OHC-related active amplification of the probe response, then the largest changes in rCM following the trauma should occur at the smallest *f*_ST_/*f*_PT_ ratios. Instead, all rCM levels decreased by a similar amount, independent of the *f*_ST_/*f*_PT_ ratio. These results suggest that rCM depends more heavily on the changes in active amplification of the ST (rather than the PT) response. In the next section, we explore this idea further using a phenomenological model of CM generation.

### Modeling CM

#### Model description

To explore the mechanisms underlying the sensitivity of rCM to acoustic trauma we developed a simple phenomenological model of CM generation in the chinchilla. In the model, the CM at the round window is calculated as a vector sum of individual CM sources (i.e., hair cells) distributed along the BM. It is assumed that the source excitation is controlled by the local BM displacement via the hair-cell transducer function (He et al., 2004; Cheatham et al., 2011). Published BM data from four different chinchilla cochleae were used to introduce some realistic intersubject variability into the model predictions. For simplicity, the CM and rCMs were calculated for one PT frequency and two ST conditions (ST1 and ST2.1). The effects of acoustic trauma on CM responses at the probe frequency were simulated for two locations of damage: the first centered around the CF place of the PT and the second near the CF place of ST2.1 (i.e., basal to the probe tone CF place). Predicted changes in the CM, rCM_ST1_, and rCM_ST2.1_ due to acoustic trauma were compared with experimental data at the appropriate PT frequency.

**Figure 4:**
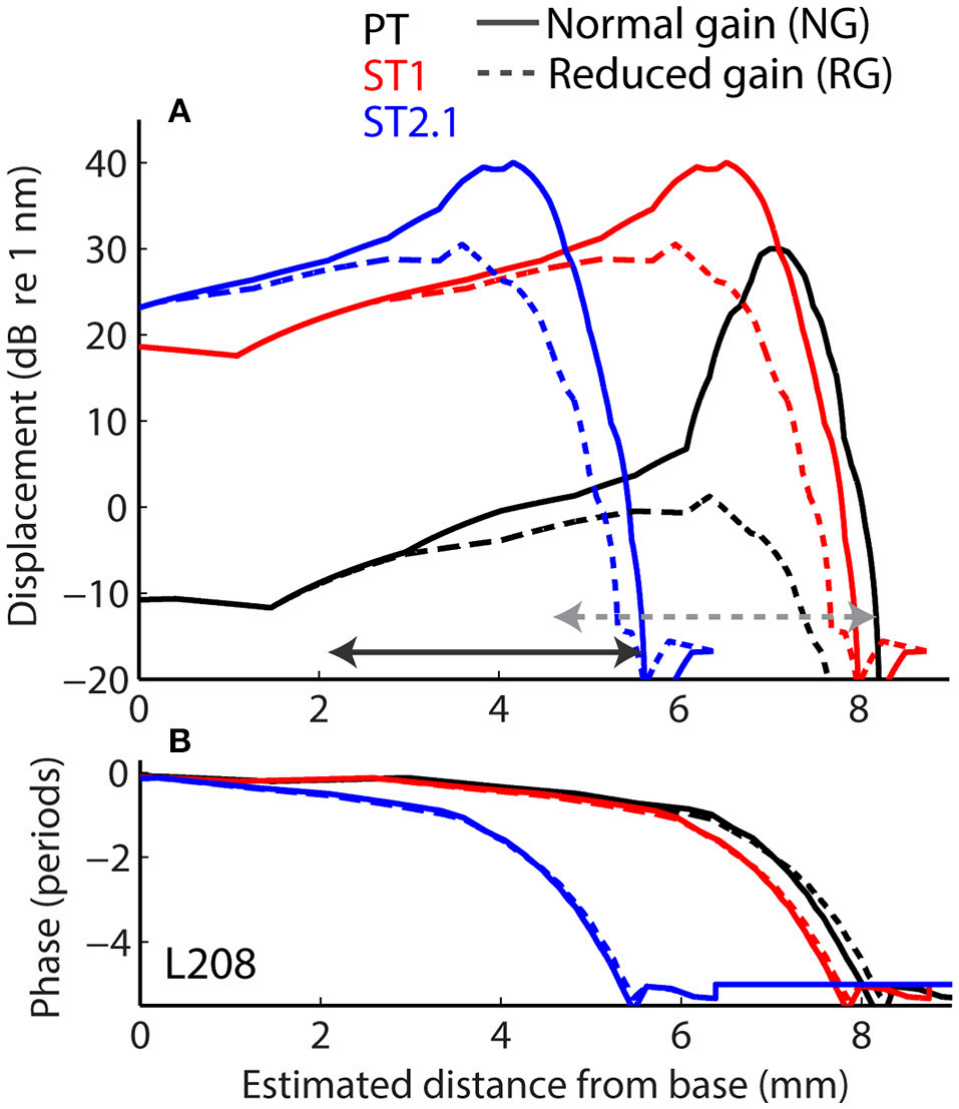
**Longitudinal basilar-membrane displacement profiles for probe tone (PT, black), ST1 (red), and ST2.1 (blue) derived from measurements in chinchilla L208 (Ruggero et al., 2000)**. The displacement profiles used to calculate CM responses for the normal cochlea are plotted with solid lines, while profiles with reduced mechanical gain are shown with dashed lines (magnitudes and phases are shown in **A** and **B**, respectively). The horizontal arrows span BM locations (1.4 octave range) where transduction was assumed damaged (in addition to gain reduction). The gray dotted arrow shows the region of damage centered at the CF place of the probe; the solid black arrow shows the damage located basal to the probe CF place (centered at ST2.1 CF place). The range of damaged locations was chosen to match the frequency range where average CAP thresholds were elevated by at least 20 dB (Figure 3A).

Longitudinal BM displacement profiles were derived from published chinchilla data obtained at a single location (CFs from 6.6 to 10 kHz) under the assumption of scaling [data from Rhode (2007) for chinchillas N92 and N157, from Ruggero et al. (1997) for L113, and from Ruggero et al. (2000) for L208]. All derived displacement profiles (magnitudes and phases) were interpolated to a resolution of 2.4 μm over a BM length of 10 mm. (For comparison, the width of a single hair cell is about 10 μm.) The probe-tone displacement profiles derived from BM responses to 30 dB SPL tones were translated using the frequency-position map (Müller et al., 2010) so that they peaked at the 4-kHz CF place (i.e., at *x* = 7.2 mm, Figure 4, solid black). Although we fixed the probe-tone frequency at 4 kHz for simplicity, model predictions can be compared to data obtained at other frequencies using scaling. The ST displacement profiles, derived from the BM responses to 60 dB SPL tones, were translated to peak at the cochlear location tuned to either 4.4 kHz (*x* = 6.8 mm; to simulate the ST1 condition, Figure 4 solid red) or 8.4 kHz (*x* = 4.5 mm; to simulate ST2.1 condition, solid blue). The instantaneous BM displacement at location *x* was calculated for a duration of 25.6 ms with sampling rate of 800 kHz as:

(1) $$\begin{array}{r} d_{\text{PT}}\left(x,t\right)=A_{\text{PT}}\left(x\right)\sin\left(2\pi f_{\text{PT}}t-\varphi_{\text{PT}}\left(x\right)\right), \end{array}$$

for the PT alone condition and as:

(2) $$\begin{array}{rc} d_{\text{PT}+\text{ST}}\left(x,t\right)=A_{\text{PT}}\left(x\right)\sin\left(2\pi f_{\text{PT}}t-\varphi_{\text{PT}}\left(x\right)\right)\\ + & A_{\text{ST}}\left(x\right)\sin\left(2\pi f_{\text{ST}}t-\varphi_{\text{ST}}\left(x\right)\right), \end{array}$$

for the PT + ST conditions, where *A* and φ represent BM displacement amplitude and phase at location *x* in response to stimulation with PT (Figure 4, black) or ST (red or blue). Because the relationship between BM displacement and *in vivo* transducer nonlinearity is unknown in the chinchilla cochlea, we arbitrarily scaled the BM displacement profiles to a maximum value of 30 dB re 1 nm for the PT stimulus (Figure 4A, black). The scaling of the PT response was chosen so that it roughly matches the “threshold” of the transducer-function nonlinearity (Siegel, 2006), since a 30 dB SPL tone at CF usually corresponds to the onset of BM nonlinearity in chinchillas (i.e., for lower stimulus levels the responses typically scale linearly; Robles and Ruggero, 2001). Subsequently, the BM displacement profiles for STs were scaled to peak at 40 dB re 1 nm to reflect the compressive growth of the BM responses at the CF (assuming a growth rate of ~0.3 dB/dB; Robles and Ruggero, 2001). Additionally, we performed computations for the ST displacement profiles scaled to a maximum value of either 30 or 50 dB re 1 nm. The resulting rCMs were either lower or higher in level, respectively, but the best match with the data was obtained with STs scaled to peak at 40 dB re 1 nm (visual inspection).

The local BM responses (Equations 1, 2) were subsequently used as the input to an OHC transducer model to estimate the contribution of each “hair cell” to the CM (with an arbitrary scale). The transducer model is a second-order Boltzmann fit to experimentally measured transducer functions in mice:

(3) $$\begin{array}{r} \text{cm}\left(x,t\right)\sim G\left[d\left(x,t\right)\right]=\frac{G_{max}}{1+K_{2}\left[d\left(x,t\right)\right]\left(1+K_{1}\left[d\left(x,t\right)\right]\right)}, \end{array}$$

where *G* is the transducer conductance for input signal *d*(*x, t*) (Equations 1, 2), *G*_*max*_ is the maximum conductance, and cm(*x, t*) is the local contribution of a hair cell's receptor current to the total CM in the time domain (Kros et al., 1995; Lukashkin and Russell, 1998; Siegel, 2006). The equilibrium constants *K*_1_ and *K*_2_ were set as in Siegel (2006), who used this model to describe properties of the CM and OAEs in chinchillas:

(4) $$\begin{array}{r} K={\text{e}}^{-\alpha \left(\frac{d\left(x,t\right)}{\beta }-1\right)}, \end{array}$$

where α_1_ = 1.56 (dimensionless), β_1_ = 24 (nm) and α_2_ = 0.656, β_2_ = 42 (nm) for *K*_1_ and *K*_2_, respectively.

The local CM source excitation at the probe-tone frequency, CM(*x, f*_PT_), was found by computing the probe-frequency Fourier component of cm(*x, t*) for a given stimulus condition (Equations 1, 2). An estimate of the conventional CM at the RW was then calculated as the vector sum of the local sources along the length of the BM in response to the probe-tone stimulus (Equations 1, 3):

(5) $$\begin{array}{r} \text{CM}\left(f_{\text{PT}}\right)=\;\Sigma w\left(x\right)\text{C}{\text{M}}_{\text{PT}}\left(x,f_{\text{PT}}\right). \end{array}$$

where, *w*(*x*) is a weighting function that controls the electrical attenuation with distance from the source. We used $w\left(x\right)=\;e^{-x\frac{A}{20\text{lo}{\text{g}}_{10}\left(e\right)}}$ with attenuation rate *A* in dB/mm. The rCM at the probe frequency was calculated as the vector difference between the summed CM source responses derived for the PT-alone and PT + ST conditions (Equations 2, 3):

(6) $$\begin{array}{rc} \text{rCM}\left(f_{\text{PT}}\right)=\;\Sigma w\left(x\right)\text{C}{\text{M}}_{\text{PT}}\left(x,f_{\text{PT}}\right)\\ - & \;\Sigma w\left(x\right)\text{C}{\text{M}}_{\text{PT}+\text{ST}}\left(x,f_{\text{PT}}\right). \end{array}$$

Because the probe frequency was fixed across all measurement conditions and only relative changes were evaluated (e.g., due to loss of gain), we initially ignored any effects of electrical source attenuation with distance (i.e., *A* = 0 dB/mm; Section Model results). Because the electrical space constants in the chinchilla cochlea are unknown, we then evaluated attenuation effects separately using a range of hypothetical attenuation rates (Section Effects of Electrical Attenuation).

Acoustic trauma was modeled as a reduction of cochlear mechanical gain at the affected location, either with or without diminishing the transduction currents (Equation 3). While mechanical gain and OHC transduction are tightly linked in a living cochlea, we do not know the exact relationship between the two variables in the chinchilla ear, and we therefore modeled them independently. To simulate reductions of mechanical gain, the BM responses to 80 dB SPL tones (from the corresponding cochlea) were used to create scaled-down displacement profiles for the probe and ST stimuli (Figure 4, the dashed lines). In these cochleae, the mechanical gain decreased by 36 to 41 dB (mean 36.4 dB, SD 4.1 dB) with increasing stimulus levels from 30 to 80 dB SPL (Ruggero et al., 1997, 2000; Rhode, 2007), values that are similar to the loss of CAP sensitivity observed in our sample (Figure 3, red). To simulate changes in transduction following the trauma, we decreased the maximum conductance by either 50% (*G*_max_ = *G*_max_/2 in Equation 3) or 100% (*G*_max_ = 0) in the affected region (see horizontal arrows in Figure 4), in addition to reducing the mechanical gain. The results were qualitatively similar, and thus only the results with *G*_max_ = 0 are discussed further.

The acoustic trauma was modeled to affect one of two cochlear locations: damage localized around the CF place of the probe tone and damage localized near the CF place of the ST2.1 (i.e., basal to the probe's CF place; see the horizontal arrows, dashed and solid, respectively, in Figure 4). In the first scenario, the BM responses to the PT and ST1 are reduced (black and red dashed lines in Figure 4) but the ST2.1 response remains unaffected (solid blue). In the second scenario, the BM response to the ST2.1 is reduced (dashed blue) while the gain of PT and ST1 responses are not changed (solid black and red). These conditions are summarized in Table 1. The simulations for damage at the probe CF place can be compared to the data measured for probe frequencies of 4–6 kHz where substantial loss of sensitivity was observed (Figure 3, red). The simulations for damage occurring basal to the CF place of the probe can be compared to the results obtained for probe frequencies of ~2 kHz, as the loss of sensitivity was centered at a location with CF about an octave above that of the probe frequency (Figure 3, red). Note that it was computationally easier to “move” the location of the damage relative to the probe CF place than it was to fix the location of the damage and compute various frequency conditions. In a scaling symmetric model, this distinction is irrelevant; in chinchillas, approximate scaling symmetry holds at CFs of 2 kHz and above (Temchin et al., 2008).

**Table 1:** **The BM displacement profiles used for calculating the CM and rCM (Equations 1–3, 5, and 6) responses across different modeling conditions**.

	**CM**	**rCM_ST1_**	**rCM_ST2.1_**
Normal	PT: NG	PT: NG, ST1: NG	PT: NG, ST2.1: NG
Gain loss at the CF place	PT: RG	PT: RG, ST1: RG	PT: RG, ST2.1: NG
Gain loss basal to CF place	PT: NG	PT: NG, ST1: NG	PT: NG, ST2.1: RG

*The BM displacement profiles for PT and ST stimuli are listed using the same key as in Figure 4; e.g., the code “PT: NG, ST2.1: RG” indicates that a normal-gain BM profile for the probe stimulus and a reduced-gain BM profile for the ST2.1stimulus were used in the calculations*.

The model is derived from real cochlear data obtained in a group of animals different from the ones used in this study. Consequently, we did not attempt to optimize the model parameters to fit our data. Our goal was to evaluate whether a model derived from realistic cochlear responses can explain the data qualitatively. Thus, no statistical testing was performed.

#### Model results

**Figure 5:**
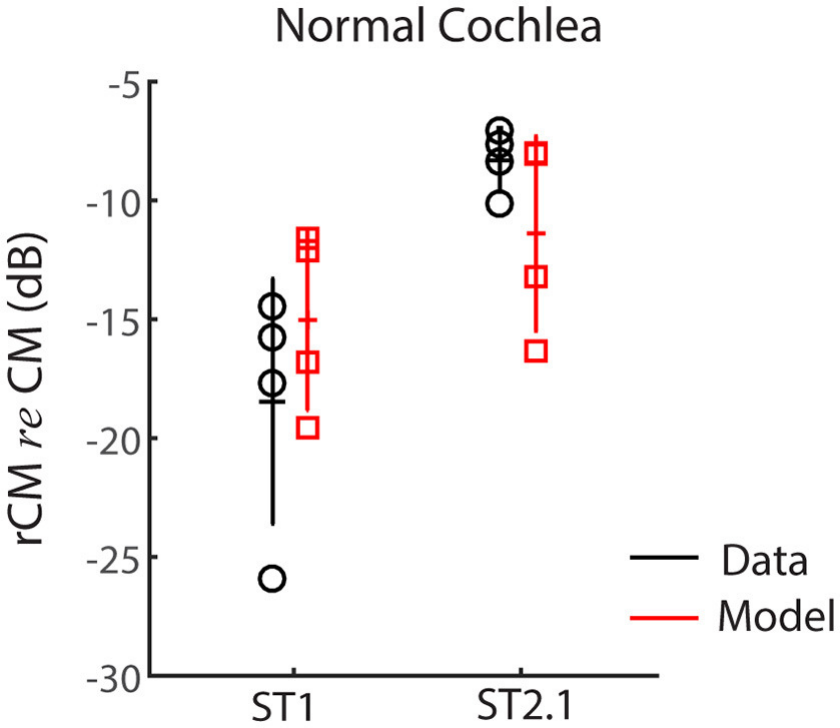
**The rCM levels (***re*** CM) obtained for a probe tone of 4 kHz for the ST1 and ST2.1 conditions for four animals**. Empirical data are shown in black and the results of CM modeling in red (normal gain, Table 1). The errors bars represent means and ± 1*SD*.

First, we evaluated whether the model captures basic properties of the CM and rCM in the normal cochlea. Figure 5 shows modeled rCM levels (*re* conventional CM) for the ST1 and ST2.1 conditions (red squares) together with the CM data obtained in our sample of animals (black circles). The model correctly predicts that rCM levels for both ST conditions fall below the levels of conventional CM (i.e., note negative *y*-axis). In the model, the ST interacts only with a subpopulation of CM sources excited by the PT, and thus rCM is always lower in level than the conventional CM. Because the phase of the local CM sources excited by PT follows the phase of the BM displacement, the model also predicts that rCM_ST1_ tends to be lower in level than rCM_ST2.1_ due to destructive interference between the CM sources located near the probe CF place (e.g., see Figure 4B, black curve).

**Figure 6:**
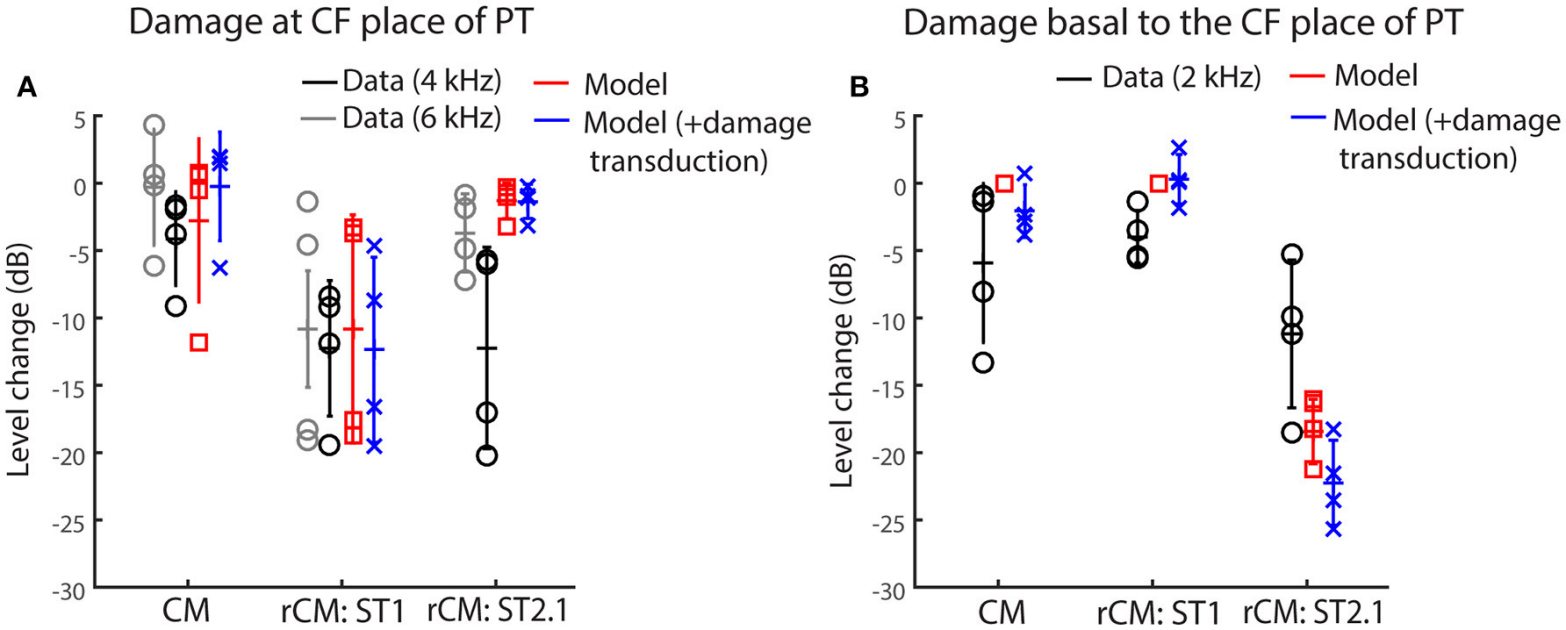
**Changes in the modeled CM and rCM (red) due to reduced cochlear gain at the probe CF place (A)** or basal to it **(B)** compared to the measured responses at frequencies that fit the model assumptions best (black and gray, see legend and text). The acoustic trauma model was expanded to include the possible loss of transduction in the regions with reduced mechanical gain (in blue).

Figure 5 shows the changes in modeled CM and rCMs resulting from different acoustic trauma conditions (red squares and blue crosses), together with corresponding chinchilla data (black and gray circles). When the gain of the BM displacement was reduced at the probe CF place (with transduction intact), the CM response either decreased or did not change much (Figure 6A, red), as the CM sources in that region tend to interfere destructively due to steep BM phase rotation (Figure 4B, black). This result agrees well with the data obtained at either the 4 or 6 kHz probe frequencies (black and gray), where at least 30 dB loss of sensitivity was observed (Figure 3, red, see the down pointing arrows). The modeled rCM_ST1_ response decreased in level following the gain reduction at the probe CF place, as also observed in the data (Figure 6A). On average 1 dB of BM gain loss produced ~0.3 dB shift in rCM_ST1_ (range 0.1–0.6 dB), similar, albeit less, than typically observed in the data (see α listed in Figures 2E–H). The shift in rCM_ST1_ level following the gain reduction at probe CF place is consistent with the ST1 interacting with a small population of sources in the affected region. However, it is not known whether the decrease in rCM_ST1_ level results from decreased BM response to the PT or ST1 or both. To tease these two factors apart, we performed additional simulations where only the gain of one or the other response was changed (e.g., PT: normal gain and ST1: reduced gain vs. PT: reduced gain and ST1: normal gain). There was a tendency for the reduced-gain ST1 only condition to cause a larger decrease in the rCM_ST1_ level compared to a reduced-gain PT only condition (by 1–5 dB), but neither resulted in changes as large as the combined condition (i.e., PT: RG and ST1: RG). This suggests that the change in the rCM_ST1_ due to trauma at the probe CF place depends on both the reduced BM responses to the probe and the ST1, although the latter appears to be more critical (i.e., the lower the ST response the less its ability to saturate the local CM sources).

In contrast to rCM_ST1_, the modeled rCM_ST2.1_ was relatively unaffected by the damage at the probe CF place (Figure 6A, red). This is expected if the ST interacts with the CM sources located near its own CF place. The data obtained for probe tone at 6 kHz (Figure 6A, gray) agree well with the model predictions. However, for the 4-kHz probe tone the rCM_ST2.1_ (black) showed larger changes than predicted by the model (particularly so for the two animals, F13 and F28). This could be explained by the fact that in the model the reduction in gain was limited to the CF region of the probe tone, without affecting the CF place of the ST2.1 (Figure 4, dashed black and solid blue). In contrast, in the data the CAP thresholds were elevated over a broader frequency region affecting the ST2.1 frequency (8.4 kHz, Figure 4, red; in individual data for the F13 and F28 animals CAP shifts exceeded 20 dB, Figures 2B,C, respectively). Thus, the 4-kHz data do not match the model assumptions as well as the 6-kHz data where there was still a significant sensitivity loss at the PT frequency (~30 dB on average; Figure 4, red) but there was little change in the CAP thresholds at the ST2.1 frequency (12.6 kHz, ~5 dB on average). In conclusion, the model predicts correctly that the rCM_ST2.1_ levels remain relatively unaffected when the loss of gain is localized to the probe CF region. Including the loss in transduction currents near the probe CF place in the simulations (Figure 4A, gray dashed arrow) did not affect the agreement between the model predictions and the data (Figure 6A, blue).

When the BM gain was reduced near the CF place of ST2.1 (e.g., basal to the probe CF place), the model predicted no change in either CM or rCM_ST1_ levels (Figure 6B, red, also see Table 1), unless the damage to transduction was added to the trauma simulations (blue). Decreased transduction in the basal region (Figure 4A, black horizontal arrow) produced no consistent change in the modeled CM and rCM_ST1_ (Figure 6B, blue), while either no change or decreases were predominately seen in the data (black). These results suggest either that our overexposure paradigm affected the transduction mechanism or that our simplified model does not capture the mechanism and/or the full extent of such damage (Patuzzi et al., 1989a; Nakajima et al., 2000). In contrast, the modeled rCM_ST2.1_ levels decreased by ~20 dB following the gain reduction basal to the probe CF place (Figure 6B, red). Similar, albeit smaller, changes in the rCM_ST2.1_ levels were observed in the data (Figure 6B, black). However, as seen in the data, 1 dB of BM gain loss produced ~0.5 dB shift in rCM_ST2.1_ level (for the data see α listed in Figures 2E–H). Even larger decreases were observed when transduction was impaired as well (blue).

Altogether, our modeling results support the hypothesis that rCM is dominated by contributions from sources located near the CF place of the ST in the cochlea. Furthermore, the model implies that the sensitivity of the rCM to a local gain reduction is dictated predominantly by the decreased gain of the BM response to the ST rather than to the probe-tone stimulus. This is best demonstrated by the results for the ST2.1 condition: Even a small reduction in the BM response to ST2.1 (e.g., Figure 4, solid vs. dashed blue) diminishes the ability of the ST to drive the local CM sources into saturation. As a result, the rCM_ST2.1_ decreases in level even though there is no change in the excitation of the sources evoked by the PT (Figure 4B, Table 1).

#### Effects of electrical attenuation

**Figure 7:**
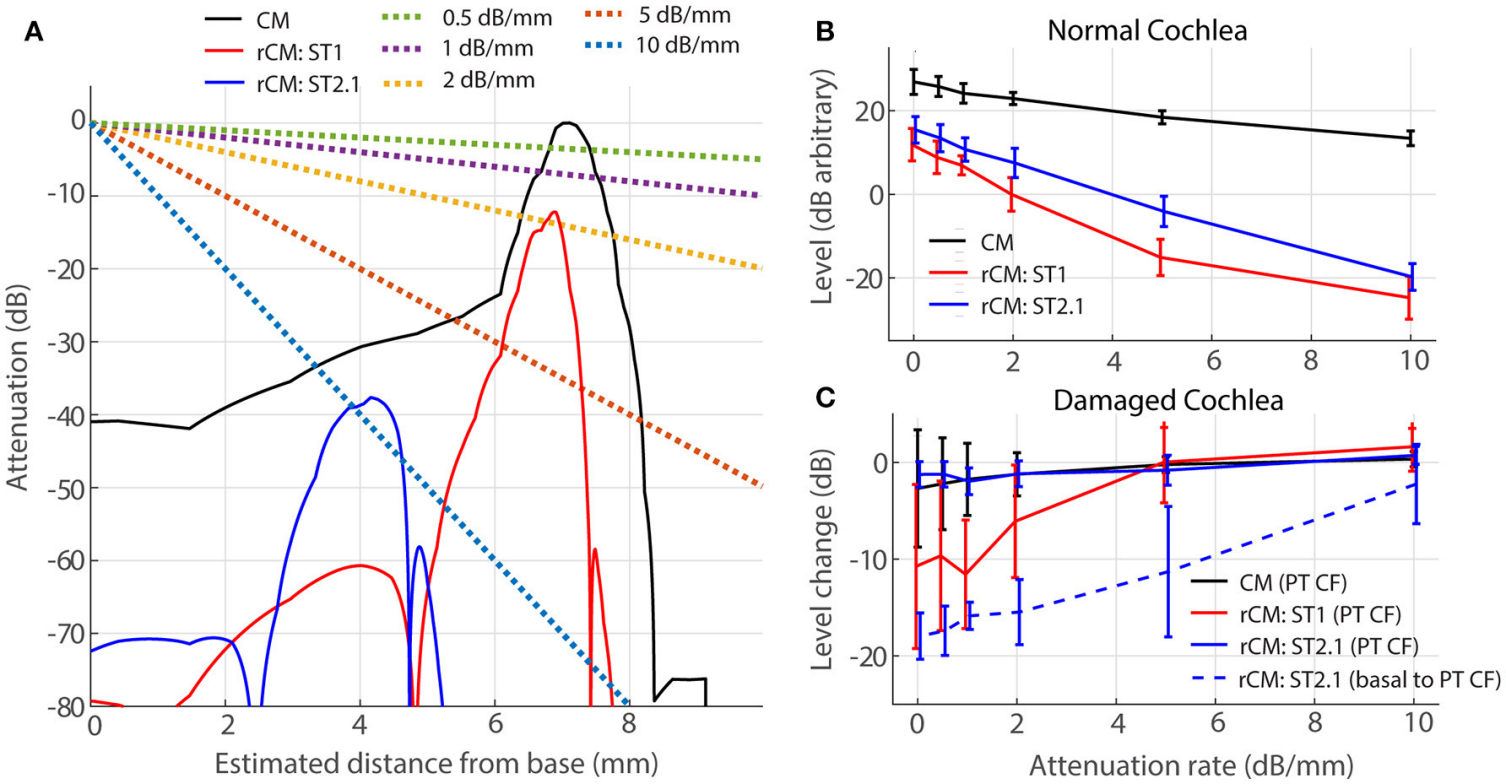
**Effects of electrical attenuation on modeled CM and rCM for a PT frequncy of 4 kHz**. Panel **(A)** shows attenuation functions *w*(*x*) for different attenuation rates (**A**, see the legend) using dashed lines, together with examples of spatial distrubutions of CM and rCM sources scaled *re* CM source strength at the CF place of the PT (solid lines; phase is not shown). The spatial source distributions were computed for normal-gain conditions based on the BM displacement shown in Figure 4. Panel **(B)** shows average levels (± 1*SD*; *n* = 4,) of CM and rCM for varying attenuation constants (*x*-axis). In **(C)** the change in CM and rCM levels due to gain reduction either at the CF place of the PT (solid) or basal to it (dashed; CM, and rCM_ST1_ are not shown here, as neither is affected by basal damage; Figure 6, red squares).

For a source at given cochlear location, the voltage recorded at the electrode decays approximately exponentially with distance between the source and the electrode (von Békésy, 1951). Thus, for an electrode placed on the RW, contributions from remote sources (i.e., at the cochlear apex) are attenuated relative to those from nearby sources (i.e., at the cochlear base). If the attenuation with distance is strong, the sensitivity of rCM to changes in cochlear gain at more apical locations may be reduced. We evaluate possible effects of electrical attenuation on rCM and CM in the model by weighting the source contributions along the cochlear length with an exponential decay function [*w*(x) in Equations (5) and (6)]. Because electrical space constants in the chinchilla cochlea are unknown, we present modeling results for several plausible attenuation rates (varied from 0.5 to 10 dB/mm). The resulting weighting functions are shown in Figure 7A (dotted lines) together with illustrative spatial distributions of CM (black) and rCM (ST1: red; ST2.1: blue) sources (phase omitted). As an example, the figure can be interpreted as follows: for *A* = 1 dB/mm, a single CM source located at the CF place of the PT (*x* = 7.2 mm) is attenuated by an additional 7 dB compared to a source located at the base (*x* = 0 mm).

In a normal cochlea, increasing the attenuation rate decreases the levels of either rCM more rapidly than it decreases the CM level (red and blue vs. black in Figure 7B). Thus, for higher attenuation rates (e.g., 5 dB/mm) the model predicts that rCM levels fall 33 and 22 dB on average below the CM level for ST1 and ST2.1, respectively. This contrasts with our experimental data, where rCM_ST1_ and rCM_ST2.1_ levels were only 18 and 8 dB lower on average than the CM level, respectively (Figure 5, black). Thus, the use of lower attenuation rates (i.e., less than ~2 dB/mm) results in more realistic model predictions. The complex electroanatomy of the cochlea likely resulted in an attenuation rate at the low end of this range.

In a damaged cochlea, the sensitivity of CM and rCM to gain reduction tends to decrease at attenuation rates above 2 dB/mm (Figure 7C). These effects are most prominent for rCM_ST1_ and damage at the CF place of PT (solid red) and for rCM_ST2.1_ and damage basal to CF place of PT (dashed blue). For instance, on average the rCM_ST1_ level was little affected by the acoustic trauma when the sources were weighted using an attenuation rate of 5 dB/mm or greater (i.e., the sources near the CF place of PT were attenuated by additional 35 dB or more and contributed little to RW signal; Figure 7A). This contrasts with the experimental data where rCM_ST1_ level was reduced by 12 dB on average following the acoustic trauma (Figure 6A, black). For moderate attenuation rates (i.e., less than ~2 dB/mm), the model predictions are not altered much relative to the zero-attenuation case, consistent with our initial assumptions.

## Discussion

### CM in assessing the functional state of the OHCS

Cochlear microphonic measurements have been used clinically mostly as an aid to differential diagnosis (e.g., in auditory neuropathy). However, CM could provide additional (e.g., place-specific) information on OHC health and function (Ponton et al., 1992; Chertoff et al., 2014). For instance, Chertoff and colleagues proposed a technique for detecting cochlear regions with missing OHCs by monitoring the level of CM evoked with a high-level 733-Hz tone-burst embedded in a high-pass masking noise. They hypothesized that the CM level should continue to increase as the cutoff frequency of the masker increased, until the noise frequency reached the region of missing OHCs. While this method is a promising approach for overcoming poor place-specificity of the CM, it does not appear sensitive enough to detect notch-type lesions in the middle cochlear turn or lesions in the apical end. Another approach for deriving place-specific information from CM was proposed by Ponton et al. (1992). In this study, a high-pass noise was used to mask basally located sources, ostensibly exposing the CM that originated at more apical locations. However, the assumptions of the method have not been validated experimentally, and it is not known whether the method provides a sensitive indicator of local damage to OHCs.

In the current study, we demonstrated that the residual CM (rCM) can successfully detect a frequency-specific elevation in neural thresholds most likely resulting from OHC impairment (Figures 2E–H, 3B). Our results suggest that rCM offers good place-specificity and sensitivity to changes in OHC-dependent cochlear gain, as measured using CAP thresholds. Importantly, though, CAP threshold measurements are not themselves free of limitations: the use of tone-burst stimuli and high levels of stimulation (necessary post-trauma) degrade the place-specificity of the CAP due to spectral splatter and spread of excitation, respectively (Özdamar and Dallos, 1978). Thus, it is likely that the CAP thresholds shifts underestimated the range and/or the degree of the cochlear sensitivity loss.

In theory, the place-specificity of the rCM is limited by the region of interaction between the PT and ST excitation patterns on the BM. The model indicates that a moderate level ST can effectively suppress sources near the peak of its own excitation pattern spanning the range of ~1–1.5 mm (i.e., ~0.4–0.6 octaves range; Figure 4; solid blue and red). In our sample, the CAP thresholds were elevated over a broader range of frequencies (Figures 2A–C, red), except for animal G03 (D) where the acoustic trauma created a sharp notch in the CAP thresholds (≥ 20 dB elevation over ~0.6 octave range). Even in this case, the change in rCM levels matched the CAP threshold elevation well, particularly for higher *f*_ST_/*f*_PT_ ratios (Figure 2H, lighter blue lines). The detection of a narrow notch in rCMs levels extracted with lower ratios (e.g., ST1 or ST1.2) can be obscured by the strong rippling pattern observed in the pre-exposure rCM levels (e.g., Figures 1B,C, dotted red). Nevertheless, the data from animal G03 suggest that rCM can detect sensitivity loss spanning a relatively narrow range of frequencies when moderate ST levels are used. The place-specificity of the rCM is likely to degrade at high ST levels due to spread of the ST excitation on the BM. In addition, place-specificity of the rCM may be diminished at low-ST frequencies due to the electrical source attenuation with distance (Section Effects of Electrical Attenuation).

Combining measurements of the rCM with conventional CM recordings may further expand the diagnostic utility of electrocochleography. Whereas, the rCM appears sensitive to changes in the active cochlear gain, the CM may be used to evaluate the state of transduction independently (e.g., Patuzzi et al., 1989a; Nakajima et al., 2000; Fridberger et al., 2002). For example, it may be possible to diagnose a loss of gain that does not depend on the OHC transduction (i.e., a mutation in the prestin protein—the motor behind the electromotility-dependent gain; Cheatham et al., 2011). Our model predicts a possible outcome of such a scenario: As illustrated in Figure 6B (red), when the acoustic trauma is simulated as a reduction in BM gain with the transduction apparatus intact, a large drop in rCM_ST2.1_ level is produced without concomitant changes in CM levels. We speculate further that the combination of these two CM measures may help to understand the mechanisms underlying other OHC-dependent phenomena, such as medial olivocochlear reflex or recovery from temporary threshold shifts (TTS). For instance, it has been suggested that recovery from TTS may involve up-regulation of the prestin protein in surviving cells as a means to compensate for the loss in gain from missing OHCs (Xia et al., 2013). In such a case, one might expect to see large changes in rCM during the recovery period with little change in CM levels. In summary, our measurements and model predictions suggest that rCM provides a unique and insightful window on the health and function of the OHCs.

### Optimal parameters for rCM measurements

The sensitivity of rCM to local changes in OHC function may depend on the stimulus parameters. In the current study, we varied one important aspect of the stimulus parameter space: the *f*_ST_/*f*_PT_ ratio. We found that rCMs mapped the frequency-range of sensitivity loss well (independent of the *f*_ST_/*f*_PT_ ratio; Figures 2E–H, 3B). However, our modeling results suggest that changes in rCMs obtained with the ST fixed at a frequency considerably higher than the PT are easier to interpret due to the spatial separation of their respective CF places in the cochlea. Using a high-frequency ST also provides the benefit of a better SNR in the mid-frequency range (at least in chinchillas; e.g., Figure 1), which may be crucial for measurements obtained using less invasive techniques (e.g., with the electrode placed on the eardrum rather than on the RW). The use of steady-state tonal stimuli, coupled with time-domain averaging and spectral analysis, presumably allows the extraction of very small signals from the noise. Our model also suggests that the sensitivity of rCM to changes in cochlear gain stems primarily from its effects on the intracochlear response to the ST rather than to the probe tone. Thus, an ST of a moderate level should be used; that is, the ST level should be high enough to saturate the local CM sources but low enough that it is still within the nonlinear range of BM processing (e.g., in chinchillas ~55–80 dB SPL; Robles and Ruggero, 2001; Siegel, 2006). The use of high-level STs is also expected to diminish the place-specificity of the rCM (Section CM in assessing the functional state of the OHCs).

Although our simple model appears to match the trends observed in the data, a more realistic model that captures the interplay between OHC transduction and its effects on amplified BM motion might improve the interpretation of our results. Furthermore, modeling the whole cochlear length with propagating BM traveling waves may be crucial for assessing whether any non-local and dynamic interactions between responses to the probe tone and ST must be considered in interpreting the origin and behavior of rCM (Versteegh and van der Heijden, 2013).

### Contamination by neural responses

At low frequencies, the RW electrode signal contains phase-locked auditory-nerve action potentials (auditory neurophonics) as well as hair-cell potentials (e.g., Henry, 1995; He et al., 2012; Lichtenhan et al., 2013). Interference between the long-delay neurophonic and the short-delay CM might explain the pattern of irregular sharp peaks and notches in CM levels at low frequencies (<2 kHz, e.g., Figure 1A; note that at higher frequencies the CM microstructure appeared smoother and nearly periodic). The significant contribution of the neurophonic to the RW potential can also be demonstrated by evaluating the phase of the response. For instance, He et al. (2012) showed that in gerbils a steep phase slope of the CM at low frequencies can be abolished by application of the neurotoxin tetrodotoxin. In our sample, similar steep phase slopes were observed in the CM responses at frequencies below ~1.5–2 kHz (data not shown), suggesting significant contamination from the neurophonic. At higher frequencies, however, the CM phase was shallow, suggesting little or no contamination from the neurophonic, as expected due to the low-pass nature of neural phase-locking (Johnson, 1980; Weiss and Rose, 1988). Thus, it seems unlikely that the neurophonic contributed to the sensitivity of rCM to the acoustic trauma centered at ~4 kHz. However, to monitor OHC function at lower frequencies, it may be necessary to separate the CM and neurophonic responses (Forgues et al., 2014; Verschooten and Joris, 2014). The use of high *f*_ST_/*f*_PT_ ratios for rCM measurements may avoid the contamination from the neurophonic, given that the neurophonic originates primarily in neurons innervating the CF place of the probe tone (Henry, 1997; Lichtenhan et al., 2014).

### Electrical attenuation with distance

Due to electrical attenuation with distance, CM sources more distant from the recording electrode contribute less to the measured response than proximal ones. Thus, for an electrode placed at the RW, contributions from more apical sources are deemphasized relative to those near the base, an effect that can compromise the place-specificity of the CM (Patuzzi et al., 1989b). The use of rCM overcomes some of the limitations of poor place-specificity of the CM. Although our modeling results confirm that strong attenuation can diminish rCM sensitivity to local change in gain (Figure 7C), our data (e.g., Figure 3B) suggest that the electrical attenuation in chinchilla is not strong enough to conceal contributions from the 4-kHz CF place (~7.2 mm away from the RW). Determining whether the rCM will prove equally successful at detecting damage to more apical cochlear locations requires further research.

Although the rate of electrical attenuation with distance in the chinchilla cochlea is unknown, our modeling results suggests that the attenuation rates are relatively small (i.e., <2 dB/mm). In contrast, intracochlear measurements of electrical space constants in other species, while varying widely across studies (from 0.042 to 2 mm), all indicate considerably higher attenuation rates (i.e., ~9–200 dB/mm; von Békésy, 1951; Misrahy et al., 1958; Johnstone et al., 1966; Fridberger et al., 2004; Dong and Olson, 2013). Our data suggest that these intracochlear measurements fail to capture actual CM attenuation rates seen from the RW. For instance, if one assumes a nominal 10 dB/mm attenuation rate, CM sources at the 4-kHz place would be attenuated by 72 dB, implying that rCM_ST1_ would be small (perhaps even undetectable) and unlikely to reveal acoustic trauma at the probe CF place—contrary to our experimental results (e.g., Figure 6A, black and gray). Similarly, Chertoff et al. (2012) concluded that attenuation rates of ~9 dB/mm are too rapid to accurately predict the growth rates of the RW CM with increasing cutoff frequency of the high-pass noise in gerbil. Perhaps the attenuation rate seen at the RW differs from the rate observed intracochlearly because of the different positions of the recording and/or the reference electrodes. Although these relationships are challenging to test experimentally, models that incorporate realistic cochlear dimensions and material properties (e.g., Teal and Ni, 2016) may provide insight on how attenuation is affected by electrode position.

## Conclusions

We demonstrated that remote (e.g., RW) measurements of cochlear-microphonic potentials may serve as sensitive indicators of the reduction in OHC-dependent cochlear gain induced by acoustic trauma. By measuring the residual CM (rCM), which represents the contributions to CM potentials from hair-cell sources located near the CF place of an additional, saturating tone (ST), it appears possible to overcome the limitations of RW recordings, which are otherwise heavily weighted by contributions from sources proximal to the electrode (i.e., at the cochlear base). Our phenomenological model of CM generation and two-tone interactions indicates that the sensitivity of rCM levels to decreased cochlear gain depends on nonlinearity at the CF place of the ST rather than of the probe. This implies that using STs of high levels, so that they do not depend on cochlear nonlinearity, may yield rCMs that are largely insensitive to the loss of gain, especially for high *f*_ST_/*f*_PT_ ratios. Thus, moderate level STs may be preferred in practice. Although all rCMs, independent of the ST condition, showed similar sensitivity to acoustic trauma, in practice, higher-frequency STs (e.g., the ST2.1) offered better SNR, possibly less contamination of rCM from the neurophonic, and easier interpretation of the data (as suggested by the model). This study demonstrates the potential for using rCM to monitor the health of the OHCs.

## Author contributions

KC contributed to the design of the experiment; to the acquisition, analysis, modeling, and interpretation of the data; and drafted the manuscript. JS contributed to the design of the experiment, to the acquisition and interpretation of the data, and to the final version of the manuscript. CS contributed to the analysis, modeling, and interpretation of the data, and to the final version of the manuscript.

### Conflict of interest statement

The authors declare that the research was conducted in the absence of any commercial or financial relationships that could be construed as a potential conflict of interest.
